# Mild and moderate cardioembolic stroke patients may benefit more from direct mechanical thrombectomy than bridging therapy: A subgroup analysis of a randomized clinical trial (DIRECT-MT)

**DOI:** 10.3389/fneur.2022.1013819

**Published:** 2022-11-24

**Authors:** Jie Cao, Pengfei Xing, Xucheng Zhu, Ronghua Chen, Huaming Shao, Jinggang Xuan, Tianwei Jiang, Pengfei Yang, Yongwei Zhang, Zifu Li, Wenhuo Chen, Tianxiao Li, Shouchun Wang, Min Lou, Ya Peng, Jianmin Liu

**Affiliations:** ^1^Department of Neurosurgery, The First People's Hospital of Changzhou/The Third Affiliated Hospital of Soochow University, Changzhou, China; ^2^Department of Neurosurgery, Naval Medical University Changhai Hospital, Shanghai, China; ^3^Department of Neurology, Naval Medical University Changhai Hospital, Shanghai, China; ^4^Department of Neurology, Zhangzhou Affiliated Hospital of Fujian Medical University, Zhangzhou, China; ^5^Department of Radiology, Henan Provincial People's Hospital of Zhengzhou University, Zhengzhou, China; ^6^Department of Neurology, First Affiliated Hospital of Jilin University, Changchun, China; ^7^Department of Neurology, Second Affiliated Hospital of Zhejiang University, Hangzhou, China

**Keywords:** cardioembolic stroke, direct mechanical thrombectomy, bridging therapy, mild and moderate stroke, DIRECT-MT

## Abstract

**Background:**

The benefit of intravenous alteplase before endovascular thrombectomy is unclear in patients with acute cardioembolic stroke.

**Methods:**

We collected cardioembolic (CE) stroke patient data from the multicentre randomized clinical trial of Direct Intra-arterial Thrombectomy to Revascularize Acute Ischaemic Stroke Patients with Large Vessel Occlusion Efficiently in Chinese Tertiary Hospitals (DIRECT-MT). The primary outcome was the 90-day modified Rankin Scale (mRS) score. Five subgroups of cardioembolic stroke patients were analyzed. A multivariable ordinal logistic regression analysis analyzed the difference in the primary outcome between the direct mechanical thrombectomy (MT) and bridging therapy groups. An interaction term was entered into the model to test for subgroup interaction. The DIRECT-MT trial is registered with clinicaltrials.gov Identifier: NCT03469206.

**Results:**

A total of 290 CE stroke patients from the DIRECT-MT trial were enrolled in this study: 146 patients in the direct MT group and 144 patients in the bridging therapy group. No difference between the two treatment groups in the primary outcome was found (adjusted common odds ratio, 1.218; 95% confidence interval, 0.806 to 1.841; P = 0.34). In the subgroup analysis, CE stroke patients with an NIHSS ≤ 15 in the direct MT group were associated with better outcomes (47 vs. 53, acOR, 3.14 [1.497, 6.585]) and lower mortality (47 vs. 53, aOR, 0.16 [0.026, 0.986]) than those in the bridging therapy group, while there were no significant differences between the two treatment groups in the outcome and mortality of CE stroke patients with an NIHSS >15.

**Conclusion:**

Mild and moderate cardioembolic stroke patients may benefit more from direct mechanical thrombectomy than bridging therapy. This need to be confirmed by further prospective studies in a larger number of patients.

## Introduction

Mechanical thrombectomy (MT) combined with intravenous thrombolysis (IVT) was confirmed to be superior to IVT alone for acute ischaemic stroke (AIS) caused by large vessel occlusion (LVO) in the anterior circulation in five randomized trials in 2015 (1). Direct Intra-arterial Thrombectomy to Revascularize Acute Ischaemic Stroke Patients with Large Vessel Occlusion Efficiently in Chinese Tertiary Hospitals: a Multicentre Randomized Clinical Trial (DIRECT-MT) pointed out that the effect and safety of direct MT was noninferior to bridging therapy for acute LVO patients eligible for IVT (2).

Cardioembolic (CE) stroke accounts for almost 30% of all ischaemic strokes (3), and the incidence is increasing (4). Cardioembolic stroke is usually more destructive than the nonembolic mechanisms of stroke, especially when considering that CE LVO is more likely to cause greater cerebral ischaemia (5). Approximately 50% of LVOs are caused by thrombi from cardioembolic (CE) sources such as atrial fibrillation (6), and the histopathologic composition of CE thrombi is different from that of noncardioembolic (N-CE) thrombi (7). The efficacy of endovascular treatment in CE LVO is higher than that in N-CE LVO (6). Alteplase is more effective in CE than in N-CE patients due to the clot composition and its dimensions (8). Our previous subgroup analysis indicated that there were no obvious differences in the modified Rankin Scale (mRS) score at 90 days and safety between the direct MT and bridging therapy groups in the different stroke etiology (9). However, we have not analyzed the effect of intravenous thrombolysis prior to endovascular treatment in the different subgroups of CE LVO in our main study.

In the DIRECT-MT trial, 44.2% of all enrolled patients were identified as having cardioembolic LVO (44.6% in the direct mechanical thrombectomy group and 43.8% in the bridging therapy). This study provided an opportunity to explore the benefit of intravenous alteplase before thrombectomy for the different subgroups of cardioembolic stroke.

## Methods

### Study design and population

We performed a *post hoc* analysis of the DIRECT-MT study. The data of patients with CE stroke were extracted and analyzed in this study. Five subgroups were designed according to the original DIRECT-MT study to estimate the effect of treatment on CE stroke: age, the baseline NIHSS, the time from onset of symptoms to randomization, the occlusion location and the collateral grades (10). NIHSS ≤ 15 was defined as the mild to moderate AIS based on Cincinnati Prehospital Stroke Severity Scale (CPSSS) study while a severe stroke was defined as NIHSS > 15 (11).

### Outcome

The primary outcome was the modified Rankin scale score at 90 days (within a window of ±14 days) (12).

The secondary outcomes were the following: the percentage of patients with functional independence (mRS ≤ 2) and a favorable outcome (mRS ≤ 3) at 90 days; the NIHSS score at 24 h and at 5 to 7 days (or at discharge); death within 90 days; the percentage of patients with successful reperfusion before thrombectomy as assessed using the extended thrombolysis in Cerebral Infarction (eTICI) score on the first intracranial angiogram (13), an eTICI score ≥ 2b on the final angiogram and recanalization at 24–72 h as assessed by CTA; and the final lesion volume on CT.

The safety outcomes included the mortality at 90 days, the percentage of patients with symptomatic and asymptomatic intracranial hemorrhage according to the Heidelberg criteria (14) and large or malignant MCA infarction, and the incidence of dissection, embolization in new cerebrovascular territory and contrast extravasation.

### Statistical analysis

The baseline data are presented with descriptive statistics as appropriate. We used a multivariable ordinal logistic regression analysis to calculate the adjusted common odds ratio (acOR) for a shift in direction toward a better functional outcome on the mRS for direct MT than for bridging therapy. This method was also used for the subgroup analysis for the relationship between the baseline variables and the primary outcome. The interaction terms were entered into the models to test for interactions between treatment and the baseline NIHSS subgroups (NIHSS ≤ 15 vs. NIHSS > 15 in primary, secondary and safety outcomes).

For the relationship between the baseline NIHSS and secondary or safety outcomes, linear or logistic-regression analyses were used, as appropriate, with the same adjustments that were used for the primary outcome. We adjusted the acOR and all secondary effect parameters for potential imbalances in the major prespecified prognostic variables adapted from the original DIRECT-MT trial protocol statistical analysis plan, and these variables included age, the modified Rankin scale score before stroke onset, cerebral collateral blood-flow status, and the time from stroke onset to randomization. Analysis of variance or the corresponding nonparametric test was used for between-treatment allocation comparisons by each subgroup. We analyzed the independent effect of the baseline NIHSS on the functional outcome and mortality with multivariable ordinal logistic regression analysis adjusted for the same prespecified variables and treatment allocation.

The acORs with 95% CIs are reported. The binary outcomes were analyzed with logistic regression and are presented as the adjusted ORs with 95% CIs. The continuous outcomes were analyzed with linear regression and are presented as the adjusted β with 95% CIs.

All analyses were conducted using SAS version 9.2 (SAS Institute). All *p*-values were two-sided, with a significance defined as < 0.05.

### Role of the funding source

The trial was funded by the Stroke Prevention Project of the National Health Commission of the People's Republic of China and the Wu Jieping Medical Foundation. This work was partly supported by the Basic Research Project of the Changzhou Science and Technology Bureau (No. CJ20200111).

## Results

### Patients and outcome of CE-LVOs

**Figure 1:**
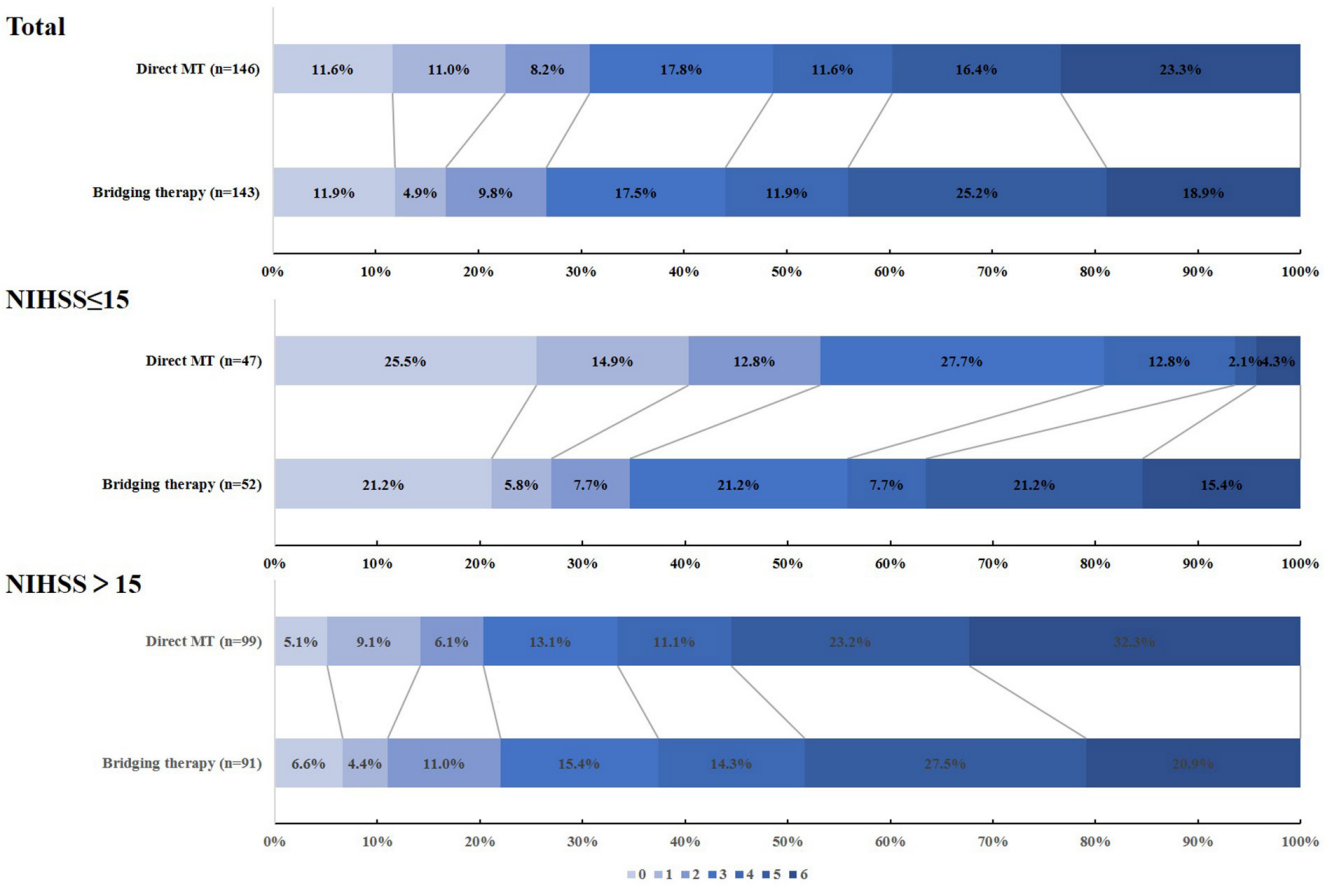
The distribution of mRS at 90 days in the direct MT and bridging therapy groups when evaluating cardioembolic stroke patients.

A total of 290 patients from the DIRECT-MT trial were regarded as cardioembolic LVOs, 146 received direct endovascular thrombectomy alone (direct MT group), and 144 underwent combination therapy with intravenous alteplase and endovascular thrombectomy (bridging therapy group). The baseline characteristics of the patients were similar in the two groups (Supplementary Table S1). The distribution of mRS at 90 days in the two groups is shown in Figure 1. For CE-LVOs, there was no obvious shift in the mRS distribution in the two treatment groups (Supplementary Table S2). The outcomes (Supplementary Table S2) between the two treatment groups for CE-LVOs have been described in our previous subgroup analysis.

### Subgroup analyses for CE-LVOs

Five subgroups for CE-LVOs were created according to the original DIRECT-MT study to explore the benefit of intravenous alteplase before thrombectomy for the treatment of CE stroke. The subgroup analyses are shown in Table 1. It was found that the adjusted common odds ratio (acOR) in the baseline NIHSS 2–15 group for functional independence at 90 days was 2.609 (95% CI, 1.074 to 6.341; *p* = 0.034). There was no heterogeneity of the treatment effect in the other subgroups, and age, the time from onset of symptoms to randomization, the occlusion location and the collateral grades were similar between the groups.

**Table 1:** Subgroup analysis of the two treatment groups for CE stroke.

**Subgroups**	**Direct MT**	**Bridging therapy**	**acOR (95% CI)**
**Age**			
18–60 years	15	14	2.003 (0.358,11.197)
60–80 years	102	101	1.519 (0.761,3.031)
≥80 years	29	29	3.591 (0.283,45.501)
**Baseline NIHSS**			
≤ 15	47	53	2.609 (1.074,6.341)
>15	99	91	1.045 (0.486,2.248)
**Time from onset of symptoms to randomization**			
≤ 171	76	72	1.633 (0.705,3.781)
>171	70	72	1.716 (0.745,3.952)
**Occlusion location**			
ICA	59	46	1.414 (0.434,4.607)
M1	65	78	1.408 (0.660,3.002)
M2	19	18	2.754 (0.329,23.030)
**Collateral grades**			
Grade 0–1	116	114	1.370 (0.697,2.692)
Grade 2–3	30	30	1.796 (0.572,5.639)

### The baseline NIHSS modifies the benefit of direct mechanical thrombectomy treatment in CE-LVO patients

In this subgroup analysis, the baseline characteristics of the patients are shown in Supplementary Table S3. 100(34.5%) of 290 CE-LVOs patients had an NIHSS ≤ 15 (47[47.0%] in the Direct MT group vs. 53[53.0%] in the Bridging Therapy group), 190 (65.5%) patients had an NIHSS > 15 (99[52.1%] in the Direct MT group vs. 91[47.9%] in the Bridging Therapy group). Patients with an NIHSS ≤ 15 had a higher median age, higher rates of occlusion of the intracranial ICA and left hemisphere, and higher levels of serum glucose. In the analysis of the NIHSS ≤ 15 group, no significant difference was found in the baseline characteristics of the patients between the treatment groups (Table 2).

**Table 2:** Baseline characteristics of the patients of the two treatment groups of NIHSS ≤ 15 CE stroke.

**Characteristic**	**Direct MT** **(*n =* 47)**	**Bridging therapy** **(*n =* 53)**	**p**
Age, *y*, median (IQR)	73 (65, 76)	71 (66, 75)	0.361
Male sex, *n* (%)	24 (51.06)	24 (45.28)	0.564
NIHSS, median (IQR)	12 (10, 14)	13 (11, 14)	0.095
Systolic BP, mmHg, median (IQR)	142 (131, 158)	142 (134, 158)	0.774
Diastolic BP, mmHg, median (IQR)	84 (74, 101)	87 (78,98)	0.78
Serum glucose, mmol/liter, median (IQR)	6.7 (5.8, 8.1)	6.6 (5.7, 7.7)	0.689
Medical history, *n* (%)			
Previous ischemic stroke	9 (19.1)	4 (7.6)	0.085
Atrial fibrillation	47 (100)	52 (98.1)	1
Hypertension	28 (59.6)	35 (66.0)	0.504
Diabetes Mellitus	8 (17.0)	9 (17.0)	0.996
**Location of intracranial artery occlusion, no./total no. (%)**			
Intracranial ICA	12/46 (26.1)	12/52 (23.1)	0.422
M1	27/46 (58.7)	36/52 (69.2)	
M2	7/46 (15.2)	4/52 (7.7)	
Hemisphere CTA, *n* (%)			
Left	35 (74.5)	39(73.6)	0.92
Right	12 (25.5)	14(26.4)	
Reperfusion before intervention (eTICI) DSA, no./total no. (%)			
0	38/45 (86.4)	40/51 (78.4)	0.215
1	0 (0)	1/51 (2.0)	
2a	6/45 (13.6)	6/51 (11.8)	
2b	0 (0)	2/51 (3.9)	
2c/3	0 (0)	2/51 (3.9)	
ASPECTS, median (IQR)	9 (8, 10)	9 (7, 10)	0.206
**Median duration (IQR), min**			
Time from stroke onset to admission	106.5 (59, 155)	120 (83, 160)	0.377
Time from stroke onset to randomization	175 (118, 206)	175 (130, 211)	0.809
Time from stroke onset to IVT	NA	183.5 (139, 224)	NA
Time from stroke onset to groin puncture	202 (155, 260)	210 (172, 252)	0.759
From hospital admission to IVT	NA	64 (49, 79)	0.198
From hospital admission to groin puncture	87 (76, 108)	88 (74, 119)	0.811
From groin puncture to revascularization	61.5 (47.5, 100)	53.5 (42, 79.5)	0.169
From hospital admission to revascularization	152 (135, 203)	151 (122, 171)	0.191

The outcomes of the patients of the two NIHSS subgroups of CE stroke are listed in Supplementary Table S4. For the primary outcome, there was a significant shift in the mRS distribution in the NIHSS ≤ 15 group between the two groups (acOR, 3.14 [1.497, 6.585]), while the mRS distribution was similar in the NIHSS > 15 group (acOR, 0.765 [0.459 to 1.275]). The interaction of the treatment-by-baseline NIHSS with the ordinal mRS distribution was obvious (*p* = 0.003) (Table 3).

**Table 3:** Treatment effect by NIHSS in CE stroke.

**Outcome**	**Measure of effect**	**Adjusted value (95% CI)**		**P-interaction**
		**NIHSS ≤ 15** **(*n =* 100)**	**NIHSS > 15** **(*n =* 190)**	
Primary outcome			
mRS at 90 days	acOR	3.14 (1.497,6.585)	0.765 (0.459,1.275)	0.003
Secondary outcomes			
Functional independence (mRS 0–2)at 90 days	OR	2.609 (1.074,6.341)	1.045 (0.486,2.248)	0.074
mRS (mRS 0–3) at 90 days	OR	5.495 (1.855,16.274)	0.923 (0.49,1.738)	0.008
NIHSS after 24 h	β	−0.891 (−4.394, 2.613)	−0.61 (−3.735, 2.514)	0.910
NIHSS at 5–7 days or discharge	β	−2.904 (−6.865, 1.057)	−0.977 (−4.838, 2.884)	0.517
Imaging outcomes	OR			
Successful reperfusion before thrombectomy, as assessed on initial DSA	OR	NA	0.146 (0.009,2.253)	0.910
eTICI score of 2b, 2c, or 3, as assessed on final angiogram	OR	1.064 (0.32,3.533)	0.803 (0.374,1.721)	0.780
Recanalization at 24–72 h, as assessed on CTA	OR	0.892 (0.192,4.138)	0.384 (0.09,1.633)	0.379
Median lesion volume on CT	β	−12.618 (-36.746,11.51)	10.488 (-16.614,37.591)	0.173
Safety outcomes
Mortality at 90 days	OR	0.16 (0.026, 0.986)	1.706 (0.861,3.377)	0.022
Serious adverse events			
Symptomatic intracranial hemorrhage	OR	NA	0.537 (0.167,1.726)	0.914
Asymptomatic intracranial hemorrhage	OR	0.559 (0.224,1.397)	0.745 (0.41,1.353)	0.608
Large or malignant MCA infarction	OR	0.614 (0.08,4.69)	1.456 (0.662,3.204)	0.450
**Procedural complication(s)**			
Dissection	OR	NA	0.979 (0.131,7.312)	0.900
Embolization in new territory	OR	1.196 (0.213,6.721)	1.198 (0.517,2.776)	0.837
Contrast extravasation	OR	NA	0.935 (0.18,4.847)	0.897
**Operation data**			
From groin puncture to revascularization	β	−12.618 (−36.746,11.51)	8.436(−6.771,23.643)	0.303
Total passes of thrombetomy	β	−12.618 (−36.746,11.51)	0.455 (−0.002, 0.912)	0.844
First pass rate	OR	3.465 (1.419,8.462)	0.741 (0.407,1.349)	0.005

For the secondary outcomes, the OR in the NIHSS ≤ 15 CE-LVO group for functional independence (mRS 0–2) at 90 days was 2.609 (95% CI, 1.074–6.341), while the aOR in the NIHSS > 15 group was 1.045 (95% CI, 0.486–2.248), and no significant treatment-by-baseline NIHSS interaction for functional independence was observed (*p* = 0.074). There was an obvious interaction between the treatment and the baseline NIHSS for mRS 0–3 at 90 days (*p* = 0.008). The aOR was 5.495(95% CI, 1.855 to 16.274) in the NIHSS ≤ 15 patients, and the aOR was 0.923 (95% CI, 0.49 to 1.738) in the NIHSS > 15 group. No significant differences were found in the other secondary outcomes in the NIHSS subgroup between the two treatment groups (Table 3).

For the safety outcomes, the differences in mortality between the Direct MT and bridging therapy groups was significant in the NIHSS ≤ 15 CE-LVOs patients (aOR, 0.16 [0.026, 0.986]), while the mortality of the two groups with NIHSS > 15 was similar (aOR, 1.706[0.861, 3.377]). The interaction between the treatment and the baseline NIHSS with the mortality was obvious (*p* = 0.022). There were no obvious differences in symptomatic intracranial hemorrhage, asymptomatic intracranial hemorrhage, large or malignant MCA infarction or procedural complications in the NIHSS ≤ 15 and NIHSS > 15 CE-LVO patients between the two treatment groups (Table 3).

Moreover, we extracted some surgical data and found that the first pass rate was higher in the direct MT group in the NIHSS ≤ 15 CE-LVO patients (aOR, 3.465[1.419, 8.462]), while the first pass rate was similar between the two treatment groups with NIHSS > 15 (aOR, 0.741[0.407, 1.349]). The treatment-by-baseline NIHSS interaction for the first pass rate was significant (*p* = 0.005). There were no significant differences in the time from groin puncture to revascularization or the total passes of thrombectomy between the two treatment groups in the NIHSS ≤ 15 and NIHSS > 15 subgroups (Table 3).

## Discussion

This subgroup analysis within the DIRECT-MT cohort evaluated the effectiveness and safety of intravenous alteplase before endovascular thrombectomy in acute cardioembolic stroke patients. No statistically significant benefit was found in the treatment of bridging therapy for CE-LVO stroke patients. In the treatment-by-subgroup analysis for CE-LVO stroke patients, we found that age, the time from onset of symptoms to randomization, the occlusion location and the collateral grades did not affect the functional outcomes and safety of bridging therapy. However, in the treatment-by-baseline NIHSS analysis, it was found that patients with an NIHSS ≤ 15 in the direct MT group had better outcomes and lower mortality rates, while the efficacy and safety remained similar in the NIHSS > 15 CE-LVO stroke patients. This finding was different from previous studies, which indicated that LVO patients who underwent bridging therapy had better functional outcomes and lower mortality than patients who underwent direct MT treatment (15, 16).

Whether to initiate intravenous thrombolysis before mechanical thrombectomy for acute LVO is still controversial. It has been found that intravenous alteplase before MT can increase the early reperfusion rate and improve functional outcomes with the hypothesis that alteplase can reduce the hardness of the thrombi and can dissolve them (17, 18). On the other hand, intravenous alteplase was reported to cause thrombus fragmentation, thrombus migration and clot formation in new territories, which may lead to longer recanalization times and lower full reperfusion rates (19). In addition, intravenous alteplase may increase the incidence of symptomatic ICH (20). Our study found that higher percentages of sICH were more likely to occur in the patients treated with bridging therapy than in the patients with direct MT (aOR, 0.37 [0.125, 1.089]; *p* = 0.071). However, no significant differences were found in the patient functional outcome, final recanalization rate or the development of an embolism in new territory between the direct MT and bridging groups.

In our study, a better functional outcome and a lower mortality rate were observed in the CE-LVO patients with an NIHSS ≤ 15 in the direct MT group, while no difference in the functional outcome and mortality was found in the CE-LVO patients with an NIHSS > 15. Furthermore, a higher first-pass reperfusion rate was achieved in CE-LVO patients with an NIHSS ≤ 15 in the direct MT group, while there was no difference between the two treatment groups in the first-pass reperfusion rate in CE-LVO patients with an NIHSS > 15. As Zaidat et al. (21) reported, first-pass reperfusion was associated with higher rates of a better functional outcome and a lower mortality rate. This interaction was also found in the ETIS (endovascular treatment in ischaemic stroke) study (22) and was identified by Nikoubashman et al. (23). Therefore, we boldly inferred that first-pass reperfusion was the main cause for the difference in the effectiveness and safety between the two treatment groups in CE-LVO patients with NIHSS ≤ 15, while there were no differences in the other factors. On the one hand, first-pass reperfusion can reduce the time from onset to recanalization and limit the expansion of the infarct core volume (24). On the other hand, repeated mechanical thrombectomy would cause more injury to the vascular endothelium, which potentially has a negative impact on the efficacy and safety (25, 26). In addition, Chueh et al. (27) reported that thousands of tiny thrombus fragments were caused by any mechanical thrombectomy, and these tiny thrombus fragments could escape to small arterioles and capillaries and occlude them. These tiny thrombus fragments cannot be seen on digital subtraction angiography and magnetic resonance imaging, but they could lead to new small embolic infarctions and modify the clinical outcome (28). Moreover, as previous studies have described, the predictors of first-pass reperfusion included age, the occlusion sites, the combined first-line device strategy and so on. However, differences in these factors were not found in the two treatment groups in the CE-LVO patients with an NIHSS ≤ 15 in our study. A possible explanation could be that alteplase works better in medium vessel occlusions (29) or cardioembolic thrombi (8). In our study, more MCA occlusion occurred in the CE-LVO patients with an NIHSS ≤ 15 than in those with an NIHSS > 15. However, a very low percentage of patients were infused with a full dose of alteplase before MT (2). We hypothesized that inadequate alteplase before MT would not only reduce the effect of intravenous thrombolysis but would also destroy the stability of the thrombus (30). The decrease in thrombus stability will increase the incidence of thrombus fragmentation and subsequent distal embolism (31, 32), further reducing the rate of successful reperfusion after the first pass and complete recanalization. A higher first-pass rate and complete recanalization were associated with better neurologic outcomes (21, 33). Although there was no difference in the final complete recanalization between the two treatments, thrombus fragmentation and small distal emboli may be important factors affecting the clinical outcome (28).

This study has some limitations. One limitation is the small sample size. In the DIRECT-MT study, approximately 45% of strokes were caused by cardioembolism, and patients with an NIHSS < 15 accounted for only approximately one-third of the CE stroke patients, with ~50 patients per treatment group. And although the overall proportion of the site of the occlusion is not different, there were M2 patients of NIHSS ≤ 15 in the direct MT group which are more prone to be recanalized by IVT and associated with better prognosis. Second, almost all cardiogenic strokes (99%) are caused by atrial fibrillation, which is a relatively higher proportion than the real world. Previous studies have indicated that IVT for stroke patients with AF provided no benefit and potentially increased the risk of intracerebral hemorrhage and mortality (34). This may partially explain our results. In addition to atrial fibrillation, cardiogenic stroke can have many other causes, including foramen ovale, aortic arch atheroma, prosthetic heart valves and others (4). These patients were found to have an undetermined cause of stroke in the DIRECT-MT study through multimodal long-term vascular and cardiac examinations (35), although the thrombus composition could be similar to CE stroke (7). We might be able to generate more widely applicable theories when that data is available. In addition, the infusion of alteplase was completed before MT in only 4 of 53 patients of NIHSS ≤ 15 in the bridging group. DIRECT-MT study included no transit patients. The patients were all randomized at the main center. If the patient was randomly assigned to the bridging group, start using rt-PA and enter the operating room at the same time for thrombectomy. This may reduce the effect of alteplase and influence the results of this study.

In conclusion, thrombectomy alone might be a more reasonable choice for mild and moderate cardioembolic stroke patients. This need to be confirmed by further prospective studies in a larger number of patients.

## Data availability statement

The raw data supporting the conclusions of this article will be made available by the authors, without undue reservation.

## Ethics statement

The study was reviewed and approved by the ethics committees and research boards of the First People's Hospital of Changzhou and Naval Medical University Changhai hospital. Written informed consent was obtained before enrollment from all the patients or their legal representatives. The patients/participants provided their written informed consent to participate in this study.

## Author contributions

YP, ML, and JL conceived and designed the study and including quality assurance and control. JC and PX collected the data and wrote the paper. PY and YZ designed the study's analytic strategy. ZL, WC, TL, and SW analyzed the data. XZ, RC, HS, JX, and TJ reviewed and edited the manuscript. All authors read and approved the manuscript.

## Funding

This work was supported by a grant (GN-2017R0001) from the Stroke Prevention Project of the National Health Commission of the People's Republic of China and by the Wu Jieping Medical Foundation. This work was partly supported by the Basic Research Project of the Changzhou Science and Technology Bureau (No. CJ20200111).

## Conflict of interest

The authors declare that the research was conducted in the absence of any commercial or financial relationships that could be construed as a potential conflict of interest.

## Publisher's note

All claims expressed in this article are solely those of the authors and do not necessarily represent those of their affiliated organizations, or those of the publisher, the editors and the reviewers. Any product that may be evaluated in this article, or claim that may be made by its manufacturer, is not guaranteed or endorsed by the publisher.
