# Supplementary material for: Mild and moderate cardioembolic stroke patients may benefit more from direct mechanical thrombectomy than bridging therapy: A subgroup analysis of a randomized clinical trial (DIRECT-MT)
Source: Front Neurol. 2022 Nov 24;13:1013819. doi: 10.3389/fneur.2022.1013819 (PMC9730510; doi:10.3389/fneur.2022.1013819)
Supplement: Supplementary file 1 [file Table_1.docx]

| Characteristic | Direct MT  （n=146） | Bridging therapy  （n=144 ） | p |
| --- | --- | --- | --- |
| Age, y, median (IQR) | 74 (66, 78) | 72 (65.5, 78) | 0.549 |
| Male sex, n (%) | 68 (46.58) | 62 (43.06) | 0.547 |
| NIHSS, median (IQR) | 18 (14, 23) | 19 (14, 23) | 0.893 |
| Systolic BP, mmHg, median (IQR) | 143 (131, 160) | 146 (134, 161) | 0.344 |
| Diastolic BP, mmHg, median (IQR) | 84.5 (77, 95) | 85 (76.5, 95.5) | 0.843 |
| Serum glucose, mmol/liter, median (IQR) | 7.1 (6.2, 8.8) | 7.3 (5.9, 8.6) | 0.663 |
| Medical history, n (%) |  |  |  |
| Previous ischemic stroke | 24 (16.4) | 24 (16.7) | 0.958 |
| Atrial fibrillation | 144 (98.6) | 143 (99.3) | 1.000 |
| Hypertension | 92 (63.0) | 97 (67.4) | 0.437 |
| Diabetes Mellitus | 32 (21.9) | 29 (20.1) | 0.71 |
| Location of intracranial artery occlusion,  no./total no. (%) |  |  |  |
| Intracranial ICA | 59/143 (41.3) | 46/143 (32.2) | 0.283 |
| M1 | 65/143 (45.5) | 78/143 (54.6) |  |
| M2 | 19/143 (13.3) | 18/143 (12.6) |  |
| Hemisphere CTA, n (%) |  |  |  |
| Left | 80 (54.8) | 79 (54.9) | 0.991 |
| Right | 66 (45.2) | 65 (45.1) |  |
| Reperfusion before intervention (eTICI) DSA,  n (%) |  |  |  |
| 0 | 125/141 (88.7) | 116/139 (83.5) | 0.371 |
| 1 | 2/141 (1.4) | 4/139 (2.9) |  |
| 2a | 13/141 (9.2) | 12/139 (8.6) |  |
| 2b | 1/141 (0.7) | 5/139 (3.6) |  |
| 2c/3 | 0(0.0) | 2/139 (1.4) |  |
| ASPECTS, median (IQR) | 9 (7, 10) | 9 (7, 10) | 0.864 |
| Median duration (IQR) , min |  |  |  |
| Time from stroke onset to admission | 115 (67.5, 149.5) | 110 (71.5, 160) | 0.571 |
| Time from stroke onset to randomization | 170 (127, 206) | 171 (124.5, 211) | 0.926 |
| Time from stroke onset to IVT | NA | 182.5 (130, 225) | NA |
| Time from stroke onset to groin puncture | 201 (160, 244) | 205 (159.5, 248.5) | 0.620 |
| From hospital admission to IVT | NA | 61(48, 78) | NA |
| From hospital admission to groin puncture | 170 (127, 206) | 171 (124.5, 211) | 0.926 |
| From groin puncture to revascularization | 66 (45, 102) | 59 (40, 85) | 0.054 |
| From hospital admission to revascularization | 158 (131, 203) | 153 (124, 181.5) | 0.188 |

Table S1 Baseline characteristics of the patients of the two treatment groups of CE stroke

| **Outcome** | **Measure of Effect** | **Direct MT**  **(n=146)** | **Bridging therapy**  **(n=144)** | **Adjusted value**  **(95% CI)** | **Adjusted p value** |
| --- | --- | --- | --- | --- | --- |
| **Primary outcome** |  |  |  |  |  |
| No. of patients with data |  | 146 | 143 |  |  |
| mRS at 90 days, median (IQR) | acOR | 4 (2, 5) | 4 (2, 5) | 1.218 (0.806, 1.841) | 0.350 |
| **Secondary outcomes** |  |  |  |  |  |
| Functional independence (mRS 0–2) at 90 days ,  no. /total no. (%) | OR | 45/146 (30.8) | 38/143 (26.6) | 1.518 (0.855, 2.698) | 0.154 |
| mRS(mRS 0–3) at 90 days , n (%) | OR | 71/146 (48.6) | 63/143 (44.1) | 1.472 (0.872, 2.484) | 0.148 |
| NIHSS after 24 h , median (IQR) | β | 16 (6, 22) | 13 (5, 21) | -0.527 (-2.903, 1.849) | 0.664 |
| NIHSS at 5–7 days or discharge, median (IQR) | β | 8 (2, 19) | 10 (3, 19) | -1.557 (-4.433, 1.319) | 0.289 |
| Imaging outcomes |  |  |  |  |  |
| Successful reperfusion before thrombectomy,  as assessed on initial DSA, no. /total no. (%) | OR | 1/141 (0.7) | 7/139 (5.0) | 0.135 (0.016, 1.12) | 0.064 |
| eTICI score of 2b, 2c, or 3,  as assessed on final angiogram, no./total no. (%) | OR | 113/138 (81.9) | 115/137 (83.9) | 0.847 (0.447, 1.603) | 0.610 |
| Recanalization at 24–72 hr,  as assessed on CTA—no./total no. (%) | OR | 114/126 (90.5) | 113/120(94.2) | 0.63 (0.236, 1.685) | 0.358 |
| Median lesion volume on CT (IQR), ml | β | 37.2(11.3,117.2) | 46.1(14.7,93.2) | 2.709 (-16.516, 21.935) | 0.782 |
| **Safety outcomes** |  |  |  |  |  |
| Mortality at 90 days, n (%) | OR | 34/146 (23.3) | 27/144 (18.8) | 1.177 (0.644, 2.15) | 0.596 |
| Serious adverse events |  |  |  |  |  |
| Symptomatic intracranial hemorrhage, n (%) | OR | 5/146 (3.4) | 12/144 (8.3) | 0.37 (0.125, 1.089) | 0.071 |
| Asymptomatic intracranial hemorrhage, n (%) | OR | 46/146 (31.5) | 56/144 (38.9) | 0.694 (0.422, 1.141) | 0.150 |
| Large or malignant MCA infarction, n (%) | OR | 22/146 (15.1) | 16/144 (11.1) | 1.289(0.627,2.653) | 0.490 |
| Procedural complication(s) |  |  |  |  |  |
| Dissection, n (%) | OR | 4/146 (2.7) | 2/144 (1.4) | 2.17(0.381,12.357) | 0.383 |
| Embolization in new territory, n (%) | OR | 19/146 (13.0) | 15/144 (10.4) | 1.165(0.551,2.46) | 0.69 |
| Contrast extravasation, n (%) | OR | 3/146 (2.1) | 6/144 (4.1) | 0.474(0.115,1.947) | 0.300 |

Table S2 Outcomes of the patients in the two treatment groups of CE stroke

Table S3 Baseline characteristics of the patients of the two NIHSS subgroups of CE stroke

| Characteristic | NIHSS≤15  （n=100） | NIHSS＞15  （n=190） | p |
| --- | --- | --- | --- |
| Age, y, median (IQR) | 72 (65, 76) | 74 (66, 80) | 0.026 |
| Male sex, n (%) | 48 (48.0) | 82 (43.2) | 0.431 |
| Systolic BP, mmHg, median (IQR) | 142 (132.5, 158) | 149 (131, 161) | 0.326 |
| Diastolic BP, mmHg, median (IQR) | 84 (77, 100) | 85 (77, 94) | 0.428 |
| Serum glucose, mmol/liter, median (IQR) | 6.7 (5.8, 8.1) | 7.4 (6.2, 9.0) | 0.002 |
| Medical history, n (%) |  |  |  |
| Previous ischemic stroke | 13 (13.0) | 35 (18.4) | 0.238 |
| Atrial fibrillation | 99 (99.0) | 188 (99.0) | 1.000 |
| Hypertension | 63 (63.0) | 126 (66.3) | 0.573 |
| Diabetes Mellitus | 17 (17.0) | 44 (23.2) | 0.221 |
| Location of intracranial artery occlusion ,  no./total no. (%) |  |  |  |
| Intracranial ICA | 24/98 (24.5) | 81/187 (43.1) | 0.005 |
| M1 | 63/98 (64.3) | 80/187 (42.6) |  |
| M2 | 11/98 (11.2) | 26/187 (13.8) |  |
| Hemisphere CTA, n (%) |  |  |  |
| Left | 74 (74.0) | 85 (44.7) | <0.001 |
| Right | 26 (26.0) | 105 (55.3) |  |
| Reperfusion before intervention (eTICI) DSA,  no./total no. (%) |  |  |  |
| 0 | 78/95 (82.1) | 163/185 (88.1) | 0.060 |
| 1 | 1/95 (1.1) | 5/185 (2.7) |  |
| 2a | 12/95 (12.6) | 13/185 (7.0) |  |
| 2b | 2/95 (2.1) | 4/185 (2.2) |  |
| 2c/3 | 2/95 (2.1) | 0 (0) |  |
| ASPECTS CT, median (IQR) |  |  |  |
| Median duration (IQR) , min |  |  |  |
| Time from stroke onset to admission | 115 (64, 159) | 111 (70, 151) | 0.928 |
| Time from stroke onset to randomization | 175 (125.5, 206.5) | 169 (127, 208) | 0.703 |
| Time from stroke onset to IVT | 185 (141, 230.00) | 181 (128, 225) | 0.612 |
| Time from stroke onset to groin puncture | 205.5 (162.5, 253) | 203 (160, 238) | 0.648 |
| From hospital admission to IVT | 64 (50, 80) | 59(47, 78) | 0.463 |
| From hospital admission to groin puncture | 88 (74, 111) | 88 (68, 112) | 0.505 |
| From groin puncture to revascularization | 59.5 (43.5, 83) | 64 (43, 96) | 0.25 |
| From hospital admission to revascularization | 152 (124, 182) | 158.5 (126, 200.5) | 0.439 |

Table S4. Outcome of the patients of the two NIHSS subgroups of CE stroke

| Outcome | NIHSS≤15(n=100) | |  | NIHSS＞15(n=190) | |
| --- | --- | --- | --- | --- | --- |
|  | Direct MT  (n=47) | Bridging therapy  (n=53) |  | Direct MT  (n=99)_ | Bridging therapy  (n=91) |
| Primary outcome: mRS at 90 days, n (%) |  |  |  |  |  |
| 0 | 12(25.5) | 11(21.2) |  | 5(5.1) | 6(6.6) |
| 1 | 7(14.9) | 3(5.8) |  | 9(9.1) | 4(4.4) |
| 2 | 6(12.8) | 4(7.7) |  | 6(6.1) | 10(11.0) |
| 3 | 13(27.7) | 11(21.2) |  | 13(13.1) | 14(15.4) |
| 4 | 6(12.8) | 4(7.8) |  | 11(11.1) | 13(14.3) |
| 5 | 1(2.18) | 11(21.2) |  | 23(23.2) | 25(27.5) |
| 6 | 2(4.3) | 8(15.4) |  | 32(32.3) | 19(20.9) |
| Secondary outcomes |  |  |  |  |  |
| Functional independence (mRS 0–2）at 90 days, n (%) | 25(53.2) | 18(34.6) |  | 20(20.2) | 20(22.0) |
| mRS (mRS 0–3) at 90 days, n (%) | 38(80.9) | 29(55.8) |  | 33(33.3) | 34(37.4) |
| NIHSS after 24 h (IQR) | 7(3,11) | 7(2,12) |  | 18(10,25) | 18(10,27) |
| NIHSS at 5–7 days or discharge (IQR) | 3(1,8) | 4(1,12) |  | 14.5(5,24) | 13(5,27) |
| Imaging outcomes |  |  |  |  |  |
| Successful reperfusion before thrombectomy,  as assessed on initial DSA, n (%) | 0(0.0) | 4(7.8) |  | 1(1.0) | 3(3.4) |
| eTICI score of 2b, 2c, or 3,  as assessed on final angiogram, n (%) | 38(86.4) | 42(85.7) |  | 75(79.8) | 73(83.0) |
| Recanalization at 24–72 hr,  as assessed on CTA, n (%) | 40(90.9) | 42(91.3) |  | 74(90.2) | 71(96.0) |
| Median lesion volume on CT (IQR) | 17.0 (3.3,44.0) | 35.8(11.6,64.8) |  | 64.7(15.7,124.3) | 65.2(17.9,101.8) |
| Safety outcomes |  |  |  |  |  |
| Mortality at 90 days, n (%) | 2(4.3) | 8(15.1) |  | 32(32.3) | 19(20.9) |
| Serious adverse events |  |  |  |  |  |
| Symptomatic intracranial hemorrhage, n (%) | 0(0.0) | 4(7.6) |  | 5(5.1) | 8(8.8) |
| Asymptomatic intracranial hemorrhage, n (%) | 11(23.4) | 18(34.0) |  | 35(35.4) | 38(41.8) |
| Large or malignant MCA infarction, n (%) | 2(4.3) | 3(5.7) |  | 20(20.2) | 13(14.3) |
| Procedural complication(s) |  |  |  |  |  |
| Dissection, n (%) | 2(4.3) | 0(0.0) |  | 2(2.0) | 2(2.2) |
| Embolization in new territory, n (%) | 3(6.4) | 3(5.7) |  | 16(16.2) | 12(13.2) |
| Contrast extravasation, n (%) | 0(0.0) | 3(5.7) |  | 3(3.0) | 3(3.3) |
| Operation data |  |  |  |  |  |
| Total passes of thrombetomy (IQR) | 2(1,2.5) | 2(1,2) |  | 2(1,3) | 2(1,3) |
| First pass rate, n (%) | 31(70.5) | 18(40.9) |  | 38(40.0) | 40(47.1) |
